# Perceived Relative Deprivation Across the Adult Lifespan: An Examination of Aging and Cohort Effects

**DOI:** 10.1177/01461672231195332

**Published:** 2023-09-05

**Authors:** Kieren J. Lilly, Chris G. Sibley, Danny Osborne

**Affiliations:** 1 University of Auckland, New Zealand

**Keywords:** relative deprivation, lifespan development, longitudinal modeling, ethnic group membership, cohort-sequential design

## Abstract

Despite being a core psychological construct for over 70 years, research has yet to examine how perceptions of deprivation relative to other individuals and/or groups *develop* across adulthood. As such, this preregistered study uses cohort-sequential latent growth modeling to examine changes in individual- and group-based relative deprivation (IRD and GRD, respectively) across the adult lifespan. Across 10 annual assessments of a nationwide random sample of adults (*N*_total_ = 58,878; ethnic minority *n* = 11,927; 62.7% women; ages 21–80), mean levels of IRD trended downward across the lifespan, whereas mean levels of GRD generally *increased* from young-to-middle adulthood before declining across late adulthood. Subtle cohort effects emerged for both constructs, although both IRD and GRD largely followed a normative aging process. Critically, the development of GRD—but not IRD—differed between ethnic groups, providing insights into how one’s *objective* status may shape *subjective* (dis)advantage over time.

Economic inequality has risen exponentially in the last 40 years, exacerbated by the global financial crisis in 2009 (Wessel, 2010) and the COVID-19 pandemic (Bowleg, 2020; Furceri et al., 2022). Critically, the rise in economic inequality has drastic implications for our health and well-being (Oishi et al., 2011; Wilkinson & Pickett, 2010), as well as social cohesion and democratic institutions (Osborne et al., 2022). Moreover, the effects of inequality are felt across the lifespan. For example, young adults are particularly affected by income inequality (Dorling et al., 2007) and unemployment (Statistics New Zealand, 2021), whereas aging populations are often dependent on government systems (e.g., welfare) and experience increased financial insecurity (Ebbinghaus, 2021). Thus, income inequality presents unique, age-related challenges across the lifespan.

Although objective forms of inequality are concerning, *subjective* experiences of inequality are often more predictive of responses than objective circumstances (Crosby, 1976; Runciman, 1966; Smith et al., 2012). Indeed, believing oneself or one’s group to be deprived relative to other individuals or groups, respectively, is an integral antecedent to various outcomes ranging from individual health and well-being (e.g., Abrams & Grant, 2012; Beshai et al., 2017; Osborne et al., 2012) to intergroup bias and competition (Osborne & Sibley, 2013; Pettigrew & Meertens, 1995). Fiscal forms of relative deprivation, in particular, *explain* the association between material conditions and these outcomes (e.g., Osborne, Sibley, & Sengupta, 2015) and are often more *strongly* associated with these constructs than objective circumstances (Smith et al., 2012). As such, people’s experiences of *relative* deprivation across the lifespan can provide critical insights into how they will respond to inequality.

Despite its importance, research has yet to investigate how feelings of relative deprivation develop across the lifespan. The current preregistered study addresses this oversight using 10 annual waves of data from a nationwide random panel sample to examine change (or lack thereof) in individual-based relative deprivation (IRD) and group-based relative deprivation (GRD).^1^ Using cohort-sequential growth curve modeling, we investigate whether (a) feelings of relative deprivation have changed over a 10-year period and (b) changes in relative deprivation reflect developmental change (i.e., age-related change) or cohort-based differences. Given that ethnic minorities are disproportionally affected by *objective* forms of inequality (Dhongde & Dong, 2022; Marriott & Sim, 2015), we also assess whether changes in relative deprivation differ by ethnicity. We, therefore, elucidate how feelings of relative deprivation change across the adult lifespan and how these trajectories vary by objective (dis)advantage.

## Relative Deprivation Theory

Relative deprivation theory argues that people respond to their *perceived* status rather than objective circumstances. Beginning with Stouffer and colleagues’ (1949) war-time studies, an impressive literature reveals that people’s perceptions of their status *relative* to (similar) others shape how they respond to inequality. Notably, Runciman (1966) articulated two distinct forms of relative deprivation: individuals can perceive themselves as deprived relative to other individuals (IRD) or perceive their ingroup as deprived relative to other groups (GRD). Because IRD and GRD respectively originate from interpersonal and intergroup comparisons, they predict distinct outcomes (Smith et al., 2012). For example, IRD correlates negatively with health and well-being (Beshai et al., 2017; Osborne et al., 2012), whereas GRD predicts group-based outcomes, including collective action support and intergroup bias (Abrams & Grant, 2012; Osborne & Sibley, 2013; M. C. Taylor, 2002). Critically, a meta-analysis of 26 studies assessing objective and relative deprivation revealed that the effects of relative deprivation were *larger* than objective conditions (see Smith et al., 2012). As such, acknowledging the predictive power of one’s *relative* status and the distinction between IRD and GRD is crucial to explaining how and when people respond to inequality.

Despite the wealth of relative deprivation literature, research has yet to investigate how feelings of relative deprivation change over time. Indeed, research has predominantly focused on cross-sectional and experimental findings rather than longitudinal designs. Of the few studies that *do* investigate relative deprivation longitudinally, research generally focuses on the relationships IRD and GRD have with individual- and group-based outcomes, respectively (e.g., Smith et al., 2020; Zubielevitch et al., 2020), rather than how relative deprivation *develops*. Given that one’s position in the lifespan and formative experiences affect their sociopolitical and ideological attitudes (e.g., Hammond et al., 2018; Milfont et al., 2021; Zubielevitch et al., 2023), it is critical to examine how these processes influence feelings of relative deprivation over time. Indeed, different age groups across the lifespan may report distinctly higher (or lower) feelings of relative deprivation (Suls, 2014). Likewise, particular generations may feel especially deprived due to shifting cultural norms and experiences in their formative years. These groups may thus be at greater risk for poor health and well-being outcomes (in the case of IRD) or may be more susceptible to intergroup biases and prejudices (in the case of GRD). However, these processes and how relative deprivation may change across the adult lifespan remain unexplored.

### IRD Across the Lifespan

Though largely unknown, research investigating *objective* forms of inequality and social comparison processes indirectly demonstrates that relative deprivation—particularly IRD—may change as people age. Indeed, research reveals age-related changes in *objective* income over time (e.g., Jappelli, 1999; Lim & Zeng, 2016). For example, Jappelli (1999) found that wealth accumulation in Italian households primarily occurs during early and middle adulthood and flattens before declining in late adulthood. Lim and Zeng (2016) found a similar inverted *U*-shaped association between income and age in the United States, though they also found significant generational differences in housing and financial wealth. Given that IRD is often entrenched (imperfectly) in reality (see Osborne, Sibley, & Sengupta, 2015), IRD should follow a trajectory similar to *objective* income and *decrease* as people accumulate wealth and *increase* as they leave the workforce (i.e., into retirement).

Social comparison theory also suggests that IRD should decline in middle adulthood, as the tendency to compare oneself to others decreases as people age. For example, Buunk et al. (2020) investigated the cross-sectional relationship between social comparison orientation (SCO) and age and discovered that SCO was highest among young people, decreased in middle adulthood, and increased in late adulthood. Callan and colleagues (2015) also found that older adults reported weaker tendencies to engage in social comparisons and lower IRD than younger adults. Critically, social comparisons partially mediated the relationship between age and IRD. These findings, coupled with the consensus that interpersonal comparisons are essential to perceiving oneself as deprived (Kim et al., 2018; Smith et al., 2012), suggest that IRD should be higher among young adults, decrease in middle adulthood, and increase in late adulthood (Buunk et al., 2020).

Although normative developmental processes such as these may contribute to changes in relative deprivation over time, age differences may also be due to cohort effects (i.e., differences due to the unique historical zeitgeist experienced by a particular cohort). Indeed, Suls (1982) argues that one’s past environment or experiences may shape their likelihood of engaging in social comparisons. As such, age-related change in IRD may be due to cohort-based differences rather than aging. Certainly, objective deprivation across generations has increased markedly (Chen et al., 2018), suggesting that younger generations bear the brunt of poverty and unemployment (Karonen & Niemelä, 2020; Kontopantelis et al., 2018). Moreover, New Zealand (i.e., the location of the present study) underwent significant public welfare reform from the mid-20th century onwards, which substantially increased the amount of government spending on welfare (Mackay, 2003) and the types of welfare available (Welfare Expert Advisory Group, 2022). These changes, however, have predominantly benefited older generations (i.e., the “welfare generation”; see Thomson, 2015). Coupled with the onset of the housing crisis in the early 2000s, changes in material wealth and social support differences mostly benefit older generations in New Zealand (Hughes & Cardona, 2022). Thus, while IRD should follow a normative trajectory, we expect some cohort-based differences in relative deprivation over time.

### GRD Across the Lifespan

Although GRD also emerges from social comparisons, these comparisons occur at the inter*group* level and are less associated with one’s general disposition than inter*personal* comparisons (Smith et al., 2012). Therefore, GRD should differ from IRD in its growth trajectory over time. Specifically, we expect changes in GRD over time to reflect *cohort*-based differences. While research has yet to investigate this hypothesis directly, GRD varies based on the specific intergroup environment. For example, experiencing rapid, negative social change is associated with greater GRD, particularly when this affects the trajectory of their ingroup’s status (de la Sablonnière et al., 2009, 2010). One’s *environment*, rather than normative age-related changes, should therefore be more predictive of GRD.

Several unique historical events in New Zealand have increased the salience of ethnic-based inequalities. For example, Māori (New Zealand’s indigenous people) have been historically marginalized since New Zealand’s colonization by British settlers in the 18th century (Orange, 2011). In response to the Crown’s constant erasure of Māori, political activism across several spheres over the last 70 years has revived Māori language, art, and culture (e.g., see Moon, 2009; Paterson, 2010). While this activism intended to protect Māori heritage, New Zealand’s colonial history is reflected in Europeans’ reluctance to revitalize Māori language and compensate Māori for lost lands and resources (e.g., Nicholson & Garland, 1991; Sibley et al., 2005). These events highlight tensions between indigenous and settler ethnic groups in New Zealand that may uniquely affect one’s perceptions of group inequities in New Zealand.

Changes to immigration policy over the last 70 years also have created tensions between ethnic groups. Specifically, immigration policies post-World War II shifted in response to labor shortages (Ongley & Pearson, 1995), substantially increasing the Pasifika population. Subsequent changes to the 1987 Immigration Act further increased ethnic diversity in New Zealand, particularly from Asian countries, with the early 1990s experiencing an immigration “boom” (Ongley & Pearson, 1995). Today, Asian and Pasifika people represent the third and fourth largest major ethnic groups in New Zealand, respectively (Statistics New Zealand, 2018), and experience greater disadvantages relative to New Zealand Europeans, including higher discrimination rates (Houkamau et al., 2017; Talamaivao et al., 2020), greater unemployment and material hardship (Ministry of Social Development, 2016), and lower median income (Statistics New Zealand, 2018). While the extent to which different Pacific nations share a formal colonial history with New Zealand differs from that of Māori—and while Asian ethnic groups exclusively *migrated* to New Zealand—the sociopolitical histories of these groups reveal their generally disadvantaged status relative to New Zealand Europeans. That these sociopolitical changes occurred within the same 70-year period also suggests a unique period of tensions between ethnic groups that would undoubtedly affect those who experienced them firsthand. Thus, cohort-based differences in GRD should emerge among those who experienced these events (vs those who did not).

We also expect these ethnic minority groups (relative to majority groups) to experience greater (a) mean levels of GRD and (b) linear increases in GRD. Indeed, the pathways in which *objective* social (in)equalities affect individuals across the lifespan differ between ethnic groups (e.g., Stephens et al., 2022), with ethnic minorities experiencing greater rates of unemployment and financial insecurity (Iceland, 2019; Marriott & Sim, 2015; Pager & Shepherd, 2008). Given the persistent increase in income inequality (e.g., McCall & Percheski, 2010), minority group members should experience greater increases in GRD than their majority group counterparts. That said, research has yet to investigate these processes, and feelings of GRD over time may not mirror objective circumstances (see Tiraboschi & Maass, 1998). Thus, it is critical to investigate how feelings of GRD change over time and whether these feelings differ by group status.

## Overview of the Current Study

The current study investigates changes in IRD and GRD over a 10-year period and whether these rates of this change differ between minority and majority group members. While research has yet to investigate these associations, research investigating objective forms of inequality and social comparison processes inform our hypotheses. Based on normative age-related experiences (e.g., first jobs, retirement), we expect IRD to differ across the lifespan (**Hypothesis 1a**), albeit with some cohort-based differences. Specifically, we theorize a curvilinear U-shaped association whereby IRD decreases from young-to-middle adulthood as a person accumulates wealth and tenure in the workforce. Once people leave the workforce and enter retirement, IRD should begin to increase. Given that IRD stems from inter*personal* comparisons, the change trajectories in IRD should not differ between ethnic groups (**Hypothesis 1b**). That is, minority and majority group members should show a similar trajectory in IRD over time (though ethnic minorities will likely have higher mean levels of IRD relative to ethnic majority group members, given their objective circumstances).

In contrast, cohort-based differences in GRD should be more evident than age-related changes over time (**Hypothesis 2a**) because one’s environment shapes perceptions of—and attitudes toward—group-based inequalities. Specifically, several unique historical events have occurred during the last 70 years in New Zealand that have impacted the salience of ethnic-based disparities (e.g., the Māori Renaissance and increased immigration). Because events like these should indelibly impact cohorts that experienced them while coming of age, cohort differences in GRD should emerge. Finally, minority group members should experience greater linear increases in GRD than majority group members (**Hypothesis 2b**) because feelings of GRD stem from inter*group* comparisons, and minority groups are disproportionally affected by income inequality.

We examine these hypotheses using 10 annual waves of longitudinal data from a nationwide random sample of New Zealand adults. Given that feelings of relative deprivation depend on one’s context (Smith et al., 1999), and there are considerable within-person changes in these constructs year-to-year (Lilly et al., 2023), 1-year time intervals (i.e., the shortest time interval available for our data) are the most appropriate intervals to assess feelings of IRD and GRD as people age.

**Figure 1.**
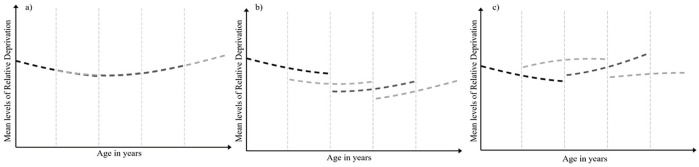
Illustrative example of aging, period, and cohort effects. *Note*. For explanatory purposes, we present four 5-year birth cohorts across 10 annual assessment occasions. Panel (a) displays an aging effect whereby the four cohorts demonstrate comparable intercepts and rates of change over time (i.e., estimates overlap considerably across cohorts). Panel (b) displays a period effect whereby cohorts differ in their initial levels of a construct but demonstrate comparable rates of change over time. Panel (c) demonstrates a cohort effect whereby cohorts differ both in their initial levels of a construct and in their rate of change over time.

To test our hypotheses, we use cohort-latent growth modeling, an analytic approach that can distinguish between aging and cohort effects (Prinzie & Onghena, 2005). We simultaneously examine rates of change over time among 11 different birth cohorts using three separate cohort-sequential latent growth models that make different assumptions about the development of relative deprivation over time (for recent examples, see Milfont et al., 2021; Zubielevitch et al., 2023). Namely, these models test whether changes in relative deprivation over time reflect (a) common developmental trends across the lifespan (i.e., an *aging* effect), (b) common trends over time across birth cohorts due to shared societal factors (i.e., a *period* effect), or (c) differences between cohorts due to different societal factors associated with the period a person was born and reached maturity in (i.e., a *cohort* effect; for an illustrative example, see Figure 1). Critically, we examine whether there are group-based differences in the trajectory of IRD and GRD over time between majority and minority ethnic groups. Such an approach allows us to determine whether the broad trends in IRD and GRD over time are distinct across groups that may have different experiences of *objective* inequality.

## Method

### Transparency and Openness

We report all sample sizes and exclusion criteria. Our hypotheses and analyses were preregistered on Open Science Framework (https://osf.io/598sf). All preregistered analyses are reported. Data were analyzed using *Mplus* v.8.8 (Muthén & Muthén, 1998–2023) and syntax for our analyses is available at https://osf.io/75snb/. The overall New Zealand Attitudes and Values (NZAVS) project was approved by the University of Auckland Human Ethics Committee and is renewed every three years. The data presented in this study are not publicly available due to restrictions imposed by the Ethics Committee. However, a deidentified data set containing the variables analyzed here is available upon request from the authors for the purpose of replication. Sibley (2021) provides further details of the sample procedures, retention rates, measures, and ethics approvals for the NZAVS.

### Sampling Procedure

We analyzed data from the NZAVS—a longitudinal probability study of adults that began in 2009. Participants for the NZAVS were initially randomly sampled from the electoral roll (Time 1; *N* = 6,518, response rate 16.6%). To address sample attrition and diversify the sample, five booster samples were conducted at Time 3 (2011; booster *n =* 2,966), Time 4 (2012; booster *n =* 5,107), Time 5 (2013; booster *n =*7,579), Time 8 (2016; booster *n =* 7,667), and Time 10 (2018; booster *n =* 29,293). By Time 12, 69,021 participants had completed at least one wave of the study, with good wave-to-wave retention (67.9%–85.6%).

Because we use data from an ongoing longitudinal study, the sample size was determined by our ability to retain participants. Although the NZAVS began in 2009, we first assessed relative deprivation at Time 3 in 2011. As such, we focus on participants who provided partial (*N* = 56,678) or complete (*N* = 2,220) responses to our focal variables from Time 3 (2011) to Time 12 (2020) of the NZAVS (*N*_total_ = 58,878). The average age of the sample at Time 3 was 44.35 years (*SD* = 11.92). Of the total sample, 62.7% were women (see Table 1 for a breakdown by age), and 77.6% were born in New Zealand. As for ethnicity, participants identified as New Zealand European (79.7%), Māori (12.6%), Asian (5.0%), or Pasifika (2.7%).

**Table 1.** Age and Sample Size for Birth Cohorts by Ethnic Group Status.

Birth cohorts	Age at Time 3 (~2011)	Age at Time 12 (~2020)	Women %	Individual-based relative deprivation		Group-based relative deprivation	
				Ethnic majority *n*	Ethnic minority *n*	Ethnic majority *n*	Ethnic minority *n*
1990–1986	21	30	67.1	3,316	1,243	3,318	1,243
1985–1981	26	35	67.2	3,499	1,240	3,498	1,240
1980–1976	31	40	66.2	4,110	1,319	4,110	1,317
1975–1971	36	45	66.1	5,052	1,527	5,053	1,528
1970–1966	41	50	63.9	5,919	1,678	5,921	1,680
1965–1961	46	55	62.3	7,333	1,723	7,334	1,721
1960–1956	51	60	59.7	8,162	1,636	8,160	1,636
1955–1951	56	65	57.1	6,216	983	6,221	984
1950–1946	61	70	58.1	1,846	318	1,846	318
1945–1941	66	75	55.0	995	173	996	173
1940–1936	71	80	51.7	479	76	479	76
*n*				46,927	11,916	46,936	11,916
*N*_total_				58,843		58,852	

*Note.* Youngest age in birth cohort was used as an indication of participants’ age at Time 3. Ethnic majority = Europeans/Pākehā; Ethnic minority = Māori, Pasifika, and Asian.

We grouped participants into 5-year cohorts based on their birth year. To inspect group-based differences in these processes over time, we also examined the aging, period, and cohort effects of relative deprivation by ethnic group status (i.e., membership in a minority or majority ethnic group). Table 1 displays each birth cohort’s sample size and age by ethnicity across the 10 assessment occasions.

### Measures

IRD was measured using two items adapted from Abrams and Grant (2012): (a) “I’m frustrated by what I earn relative to other people in New Zealand”; and (b) “I generally earn less than other people in New Zealand.” The items were measured on a 1 (*strongly disagree*) to 7 (*strongly agree*) scale and were averaged at each measurement occasion (*r*s = .41–.44).

GRD was measured using two items adapted from Abrams and Grant (2012): (a) “I’m frustrated with what my ethnic group earns relative to other groups in New Zealand” and (b) “People from my ethnic group generally earn less than other groups in New Zealand.” The items were measured on a 1 (*strongly disagree*) to 7 (*strongly agree*) scale and were averaged at each measurement occasion (*r*s = .37–.49).

Minority status was dummy-coded (0 = New Zealand European, 1 = Minority).

## Analytic Approach

To examine the change trajectories of IRD and GRD over time, and whether these differed by ethnicity, we estimated multigroup cohort-sequential latent growth models based on 5-year birth cohorts (e.g., see Zubielevitch et al., 2023). Cohort-sequential analysis extends traditional latent growth curve modeling by simultaneously estimating mean growth trajectories for different cohorts. The overlapping estimates in different cohorts therefore allow us to identify common developmental trends across the adult lifespan *and* whether there are differences in IRD and GRD between birth cohorts over time (Prinzie & Onghena, 2005).

We conducted all our analyses using *Mplus* v.8.8 with full information maximum likelihood (FIML) estimates to handle missing data. Notably, FIML is more efficient than alternative approaches like listwise or pairwise deletion at utilizing all available data and outperforms alternative methods (e.g., listwise deletion) by producing unbiased parameter estimates and reducing Type 1 error rates (Enders & Bandalos, 2001). This method prevents us from systematically excluding participants and utilizes all available data in our analyses. For example, older participants may have been unable to complete later assessments due to health issues or death. Likewise, younger participants may not have completed earlier assessments due to their age. Whereas other analytic approaches would exclude these participants and introduce systematic biases, FIML allows us to retain these participants in our analyses.

We sorted our sample into 11 sequential birth cohorts based on birth year, spanning 1990 to 1936 (ages 21–80). We used the youngest possible age within each birth cohort to indicate participants’ age in 2011 (Time 3). As such, the 1990 to 1986 birth cohort reflected 10 years of change from ages 21 to 30, the 1985 to 1981 birth cohort reflected change from ages 26 to 35, and so on. Moreover, we separated each birth cohort by ethnicity to model majority and minority group trajectories separately for both IRD and GRD. However, the practical limitations of our data analyses require samples larger than each specific minority group (i.e., it is impractical to pursue these analyses with the smaller minority groups included in our data set). As such, we created a pan-ethnic minority group comprised of Māori (12.6%), Asian (5.0%), and Pasifika (2.7%) participants. While this approach does not account for the unique sociopolitical histories of different ethnic minorities, these ethnic groups all report greater disadvantages relative to New Zealand Europeans (e.g., Houkamau et al., 2017; Ministry of Social Development, 2016; Statistics New Zealand, 2018; Talamaivao et al., 2020). Nonetheless, caution is needed when generalizing our results across ethnic minority groups.

First, we estimated an aging model that allowed for the possibility that differences in relative deprivation are due to normative developmental change (see Figure 1). That is, the model assumes that a 50-year-old at Time 3 (2011) would have comparable feelings of IRD or GRD as a 50-year-old at Time 12 (2020). To do so, the model constrained the intercepts and slopes to equality across all 11 birth cohorts. To account for possible curvilinear rates of change over time, we estimated both linear and quadratic components. Models were age-centered at 45 years and conditioned by age so that we could plot the point estimates across a continuum from ages 21 to 80. The model thus allows us to identify any age-related trends in IRD and GRD across the adult lifespan.

We then estimated a period model—an intermediate model that allows birth cohorts to differ in their initial levels of relative deprivation but constrains to equality the rate of change across birth cohorts. For example, the model allows for differences between the mean levels of IRD or GRD for a 50-year-old at Time 3 (2011) and a 50-year-old at Time 12 (2020) but assumes that IRD or GRD are changing at the same rate over time (see Figure 1). As such, we constrained the slopes to equality but freed the intercepts for each cohort.

Finally, we estimated a birth cohort model to examine the possibility that generational differences uniquely affect feelings of relative deprivation over time. This model assumes the different birth cohorts differ in their mean levels *and* rates of change for relative deprivation because of the contextual factors associated with the period in which they were born (see Figure 1). As such, we freed the intercepts and the slopes for each birth cohort. As with the aging model, we conditioned the point estimates from this model by age to plot the trends for each birth cohort’s age range across the 10 annual assessment occasions.

To determine whether changes in relative deprivation over time reflect aging or cohort effects, we first inspected each model’s relative fit and parsimony using widely used model fit indices. These include the Comparative Fix Index (CFI ≥ .90; Wang & Wang, 2012), the standardized root mean square residual (SRMR ≤ .08; Hu & Bentler, 1999), and the root mean square error of approximation (RMSEA ≤ .06; McDonald & Ho, 2002). We also report the chi-square test statistic (χ²), although its sensitivity to large sample sizes renders it an impractical test of model fit (see Wang & Wang, 2012). Because these indices do not provide the nuance required to determine aging versus cohort processes (see Steiger, 2007), we use these global fit indices in conjunction with our plotted estimates to see if our results broadly display aging or cohort effects over time.

## Results

### Individual-Based Relative Deprivation

**Figure 2:**
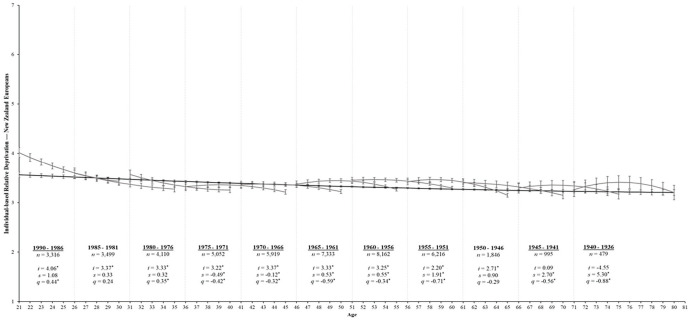
Change trajectories and comparison of aging and cohort models for ethnic majorities’ perceptions of IRD. *Note*. Change trajectories for IRD are shown by the black line from ages 21 to 80. The gray lines within each 5-year birth cohort panel demonstrate longitudinal change in IRD over 10 assessments by estimating the latent intercept (*i*), linear slopes (*s*), and quadratic slopes (*q*) and overlap with subsequent birth cohorts. Mean levels of IRD are shown on the *y* across age (in years) and annual assessments on the *x*-axis with 95% confidence intervals as error bars around each point estimate. Birth cohorts with significant rates of change over time are underlined for clarity. *p < .05.

**Figure 3.**
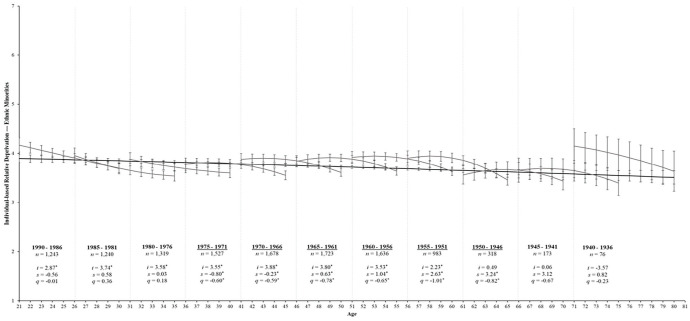
Change trajectories and comparison of aging and cohort models for ethnic minorities’ perceptions of IRD. *Note*. Change trajectories for IRD are shown by the black line from ages 21 to 80. The gray lines within each 5-year birth cohort panel demonstrate longitudinal change in IRD over 10 assessments by estimating the latent intercept (*i*), linear slopes (*s*), and quadratic slopes (*q*) and overlap with subsequent birth cohorts. Mean levels of IRD are shown on the *y* across age (in years) and annual assessments on the *x*-axis with 95% CIs as error bars around each point estimate. Birth cohorts with significant rates of change over time are underlined for clarity. *p < .05.

Table 2 shows the aging model for IRD that fits these data well, χ^2^_(1422)_ = 7,289.22, *p* < .001, CFI = .94, RMSEA = 0.04, SRMR = 0.06. The parameter estimates that best fit all birth cohorts are displayed in Table 3. For majority group members, IRD had a significant curvilinear change over time (*s* = −0.07, *SE* = 0.01, *p* < .001; *q* = 0.01, *SE* = 0.00, *p* = .026). Specifically, the black line in Figure 2 shows that IRD declined from age 21 until approximately age 50 but began to stabilize thereafter. Conversely, IRD among minority group members only exhibited a *linear* decrease over time (*s* = −0.06, *SE* = 0.01, *p* < .001; *q* = 0.00, *SE* = 0.01, *p* = .701). Indeed, the black line in Figure 3 reveals a steady decline in IRD from ages 21 to 80.

**Table 2.** Model Fit for Aging, Period, and Cohort Models.

Variable	Model	χ^2^	*df*	*p* value	CFI	RMSEA	SRMR
Individual-based relative deprivation	Aging	7,289.22	1,422	<.001	.94	0.04	0.06
	Period	6,613.25	1,402	<.001	.94	0.04	0.06
	Cohort	6,309.81	1,362	<.001	.95	0.04	0.05
Group-based relative deprivation	Aging	5,680.03	1,419	<.001	.94	0.03	0.06
	Period	5,242.61	1,399	<.001	.94	0.03	0.05
	Cohort	5,043.51	1,359	<.001	.95	0.03	0.05

*Note*. χ^2^ = chi-square; *df* = degrees of freedom; CFI = comparative fit index; RMSEA = root mean square error of approximation; SRMR = standardized root mean square residual.

**Table 3.** Parameter Coefficients for the Aging Models for Perceptions of IRD and GRD by Ethnic Group.

	Estimate	*SE*	Est./*SE*	*p* value	95% CI		Variances
Aging model					LB	UB	
Individual-based relative deprivation^a^							
Ethnic majority							
Intercept (*i*)	3.37	0.01	436.80	<.001	3.35	3.38	1.42*
Linear slope (*s*)	−0.07	0.01	−12.78	<.001	−0.08	−0.06	0.00
Quadratic slope (*q*)	0.01	0.00	2.23	.026	0.00	0.01	0.00
Ethnic minority							
Intercept (*i*)	3.76	0.02	246.05	<.001	3.73	3.79	1.42*
Linear slope (*s*)	−0.06	0.01	−6.48	<.001	−0.08	−0.04	0.00
Quadratic slope (*q*)	0.00	0.01	−0.38	.701	−0.01	0.01	0.00
Group-based relative deprivation^b^							
Ethnic majority							
Intercept (*i*)	2.09	0.01	399.90	<.001	2.08	2.10	0.51*
Linear slope (*s*)	0.03	0.00	7.19	<.001	0.02	0.03	0.00
Quadratic slope (*q*)	−0.01	0.00	−3.53	<.001	−0.01	−0.00	0.00
Ethnic minority							
Intercept (*i*)	3.90	0.02	229.75	<.001	3.87	3.94	2.03*
Linear slope (*s*)	0.01	0.01	1.16	.245	−0.01	0.04	0.17*
Quadratic slope (*q*)	−0.04	0.01	−5.38	<.001	−0.05	−0.02	0.00

*Note*. Constructs are estimated separately due to computational limitations. Ethnic majority = Europeans/Pākehā; Ethnic minority = Māori, Pasifika, and Asian. *SE* = standard error; CI = confidence interval; LB = lower bound; UB = upper bound.

a Variances constrained to equality across birth cohorts and between ethnic groups. ^b^ Variances constrained to equality across birth cohorts and within ethnic groups.

* *p* < .05.

The period model for IRD also fits these data well, χ^2^_(1402)_ = 6,613.25, *p* < .001, CFI = .94, RMSEA = 0.04, SRMR = 0.06 (Table 2). Table 4 reveals that the values for the freely estimated intercepts were generally higher for each successive birth cohort for both majority and minority ethnic groups. As such, the period model suggests that older birth cohorts had higher mean levels of IRD than younger birth cohorts, irrespective of ethnicity. Like the aging model, the period model suggests a linear decrease in IRD among ethnic majority group members over time (*s* = −0.28, *SE* = 0.01, *p* < .001), but that this rate of change slowed over time (*q* = 0.01, *SE* = 0.01, *p* = .005). Conversely, the period model revealed a linear, rather than curvilinear, decline in IRD among minority group members over time (*s* = −0.36, *SE* = 0.02, *p* < .001; *q* = 0.00, *SE* = 0.01, *p* = .734).

**Table 4.** Parameter Estimates for the Period Model for Perceptions of IRD by Ethnic Group.

Birth cohort		Ethnic majority						Ethnic minority					
		Estimate	*SE*	Est./*SE*	*p*	95% CI		Estimate	*SE*	Est./*SE*	*p*	95% CI	
						LB	UB					LB	UB
1990–1986	Intercepts	3.03	0.04	75.69	<.001	2.95	3.11	3.20	0.07	46.17	<.001	3.06	3.33
1985–1981	Freely	2.98	0.03	96.49	<.001	2.92	3.04	3.17	0.05	59.02	<.001	3.06	3.27
1980–1976	Estimated	3.09	0.02	129.15	<.001	3.05	3.14	3.38	0.04	78.34	<.001	3.30	3.47
1975–1971		3.23	0.02	167.96	<.001	3.19	3.26	3.59	0.04	100.85	<.001	3.52	3.66
1970–1966		3.37	0.02	191.28	<.001	3.34	3.41	3.84	0.03	116.06	<.001	3.78	3.91
1965–1961		3.57	0.02	198.51	<.001	3.53	3.60	4.05	0.04	112.94	<.001	3.97	4.12
1960–1956		3.70	0.02	177.50	<.001	3.66	3.75	4.23	0.04	97.04	<.001	4.15	4.32
1955–1951		3.74	0.03	137.85	<.001	3.69	3.80	4.33	0.06	71.32	<.001	4.21	4.45
1950–1946		3.81	0.04	93.53	<.001	3.73	3.89	4.32	0.09	46.74	<.001	4.14	4.50
1945–1941		3.92	0.05	72.00	<.001	3.81	4.02	4.51	0.13	36.03	<.001	4.26	4.75
1940–1936		4.05	0.07	54.51	<.001	3.90	4.19	5.01	0.18	28.27	<.001	4.67	5.36
All cohorts	Linear slope constrained	−0.28	0.01	−21.04	<.001	−0.30	−0.25	−0.36	0.02	−15.71	<.001	−0.41	−0.32
All cohorts	Quadratic slope constrained	0.01	0.01	2.81	.005	0.00	0.02	0.00	0.01	0.34	.734	−0.02	0.02

*Note.* Ethnic majority = European/Pākehā; Ethnic minority = Māori, Pasifika, and Asian. *SE* = standard error; CI = confidence interval; LB = lower bound; UB = upper bound. Variances constrained to equality across birth cohorts and ethnic groups, variance of slopes constrained to 0. Variances (*i* = 1.41*, *SE* = 0.01; *s* = 0.00, *SE* = 0.00; *q* = 0.00, *SE* = 0.00).

* *p* < .05.

Finally, the cohort model for IRD also fits these data well, χ^2^_(1362)_ = 6,309.81, *p* < .001, CFI = .95, RMSEA = 0.04, SRMR = 0.05 (Table 2). For majority group members, Table 5 reveals that 9 out of 11 birth cohorts showed significant changes in IRD over the 10-year period (*p*s < .05)—only the 1985 to 1981 and 1950 to 1941 birth cohorts had stable rates over time (*p*s > .05). The gray lines in Figure 2 display each birth cohort’s trends over the 10 annual assessments. Specifically, the youngest cohorts tended to start at higher levels of IRD and demonstrated curvilinear change over time (1990–1986, *q* = 0.44, *SE* = 0.17, *p* = .011; 1980–1976, *q* = 0.35, *SE* = 0.15, *p* = .022). The 1975 to 1971 (*s* = −0.49, *SE* = 0.11, *p* < .001; *q* = −0.42, *SE* = 0.13, *p* = .002) and 1970 to 1966 birth cohorts (*s* = −0.12, *SE* = 0.04, *p* = .006; *q* = −0.32, *SE* = 0.12, *p* = .008) showed curvilinear decreases over time. Finally, the remaining older birth cohorts exhibited linear increases over time but with a downward curve.

**Table 5.** Parameter Estimates for the Cohort Model for Perceptions of IRD by Ethnic Group.

Birth cohort		Ethnic majority					Ethnic minority				
		Est.	*SE*	*p*	95% CI		Est.	*SE*	*p*	95% CI	
					LB	UB				LB	UB
1990–1986	*i*	4.06	0.62	<.001	2.85	5.27	2.87	1.04	.006	0.84	4.90
	*s*	1.08	0.66	.101	−0.21	2.37	−0.56	1.10	.610	−2.71	1.59
	*q*	0.44	0.17	.011	0.10	0.78	−0.01	0.30	.980	−0.57	0.55
1985–1981	*i*	3.37	0.33	<.001	2.73	4.02	3.74	0.60	<.001	2.63	4.89
	*s*	0.33	0.48	.492	−0.62	1.28	0.58	0.84	.489	−1.07	2.23
	*q*	0.24	0.17	.173	−0.10	0.58	0.36	0.30	.226	−0.22	0.94
1980–1976	*i*	3.33	0.12	<.001	3.11	3.56	3.58	0.21	<.001	3.16	3.99
	*s*	0.32	0.28	.240	−0.22	0.85	0.03	0.48	.945	−0.92	0.98
	*q*	0.35	0.15	.022	0.05	0.65	0.18	0.26	.492	−0.33	0.70
1975–1971	*i*	3.22	0.02	<.001	3.18	3.27	3.55	0.05	<.001	3.46	3.64
	*s*	−0.49	0.11	<.001	−0.71	−0.28	−0.80	0.21	.001	−1.21	−0.39
	*q*	−0.42	0.13	.002	−0.68	−0.15	−0.60	0.24	.013	−1.08	−0.13
1970–1966	*i*	3.37	0.02	<.001	3.33	3.41	3.88	0.04	<.001	3.81	3.96
	*s*	−0.12	0.04	.006	−0.21	−0.03	−0.23	0.07	.001	−0.38	−0.09
	*q*	−0.32	0.12	.008	−0.56	−0.08	−0.59	0.22	.007	−1.03	−0.16
1965–1961	*i*	3.33	0.04	<.001	3.25	3.41	3.80	0.08	<.001	3.64	3.94
	*s*	0.53	0.14	<.001	0.27	0.80	0.63	0.26	.014	0.13	1.13
	*q*	−0.59	0.11	<.001	−0.80	−0.38	−0.78	0.21	<.001	−1.19	−0.36
1960–1956	*i*	3.25	0.13	<.001	3.01	3.50	3.53	0.26	<.001	3.01	4.04
	*s*	0.55	0.23	.016	0.10	1.00	1.04	0.49	.035	0.07	2.01
	*q*	−0.34	0.10	.001	−0.54	−0.15	−0.65	0.22	.004	−1.08	−0.21
1955–1951	*i*	2.20	0.28	<.001	1.65	2.75	2.23	0.70	.001	0.92	3.54
	*s*	1.91	0.35	<.001	1.21	2.60	2.63	0.86	.002	0.95	4.31
	*q*	−0.71	0.11	<.001	−0.93	−0.50	−1.01	0.27	<.001	−1.54	−0.48
1950–1946	*i*	2.71	0.63	<.001	1.45	3.97	0.49	1.56	.754	−2.58	3.55
	*s*	0.90	0.62	.157	−0.35	2.14	3.24	1.55	.036	0.21	6.27
	*q*	−0.29	0.15	.061	−0.59	0.01	−0.82	0.38	.028	−1.56	−0.09
1945–1941	*i*	0.09	1.27	.942	−2.40	2.58	0.06	3.16	.986	−6.14	6.25
	*s*	2.70	1.01	.007	0.72	4.67	3.12	2.51	.214	−1.80	8.04
	*q*	−0.56	0.20	.005	−0.94	−0.17	−0.67	0.49	.175	−1.64	0.30
1940–1936	*i*	−4.55	2.47	.065	−9.41	0.29	3.57	6.60	.589	−9.37	16.51
	*s*	5.30	1.64	.001	2.08	8.51	0.82	4.40	.852	−7.80	9.44
	*q*	−0.88	0.27	.001	−1.41	−0.35	−0.23	0.73	.754	−1.65	1.20

*Note.* Ethnic majority = Europeans/Pākehā; Ethnic minority = Māori, Pasifika, and Asian. *i =* intercept; *s* = linear slope; *q* = quadratic slope. *SE* = standard error; CI = confidence interval; LB = lower bound; UB = upper bound. Variances were constrained to equality across birth cohorts and ethnic groups; variance of slopes was constrained to 0. Variances (*i* = 1.41*, *SE* = 0.01; *s* = 0.00, *SE* = 0.00; *q* = 0.00, *SE* = 0.00).

* *p* < .05.

Turning to minority group members, only 6 of the 11 birth cohorts showed significant changes in IRD over time (*p* < .05; see Table 5). Specifically, the 1975 to 1971 (*s* = −0.80, *SE* = 0.21, *p* = .001; *q* = −0.60, *SE* = 0.24, *p* = .013), and 1970 to 1966 (*s* = −0.23, *SE* = 0.07, *p* = .001; *q* = −0.59, *SE* = 0.22, *p* = .007) birth cohorts showed curvilinear decreases in IRD over time. The 1965 to 1981 (*s* = 0.63, *SE* = 0.26, *p* = .014; *q* = −0.78, *SE* = 0.21, *p* < .001), 1960 to 1956 (*s* = 1.04, *SE* = 0.49, *p* = .035; *q* = −0.65, *SE* = 0.22, *p* = .004), 1955 to 1951 (*s* = 2.63, *SE* = 0.86, *p* = .002; *q* = −1.01, *SE* = 0.27, *p* < .001), and 1950 to 1946 birth cohorts (*s* = 3.24, *SE* = 1.55, *p* = .036; *q* = −0.82, *SE* = 0.38, *p* = .028) showed significant positive linear increases in IRD over time but with a downward curve.

The remaining three youngest (1990–1976) and two oldest (1945–1936) birth cohorts showed nonsignificant rates of IRD over time (*p*s > .05). Given the considerable variability and reduced sample size for our minority group analyses, these nonsignificant effects are worth noting. Specifically, the 1990 to 1986 birth cohort showed curvilinear decreases in IRD over time (*p*s ≥ .610), while the 1985 to 1976 birth cohorts showed curvilinear increases in IRD over time (*p* ≥ .226). The oldest 1945 to 1936 birth cohorts displayed increases in IRD over time but with a downward curve (*p*s ≥ .175).

Given that the aging, period, and cohort models all fit these data comparably, a combination of these processes likely contributes to the trajectory of IRD over time. Indeed, the aging and cohort estimates presented in Figures 2 and 3 reveal clear cohort effects for IRD between the younger and older birth cohorts for both majority and minority group members. Specifically, the younger cohorts exhibited steeper declines in IRD over time, whereas the older cohorts exhibited an inverted U-shaped trajectory. That said, estimates from the cohort models largely overlapped for both groups (i.e., the 95% error bars for each point estimate largely overlapped), and the cohort model broadly overlapped with the aging model trend, particularly among older cohorts. Thus, while there are distinct trajectories over time for the youngest and oldest birth cohorts, the trajectory of IRD among ethnic majority and minority group members largely reflects a normative aging process across adulthood (supporting Hypothesis 1a). Moreover, majority and minority group members experienced similar changes in IRD across birth cohorts, suggesting that the trajectory of IRD does not differ considerably between ethnic groups (supporting Hypothesis 1b).

### Group-Based Relative Deprivation

Regarding GRD, we again modeled the aging, period, and cohort models for both majority and minority ethnic groups. However, we noticed that constraining the variances in the intercepts and slopes to equality across ethnic groups resulted in a poor model fit, χ^2^_(1422)_ = 13,338.63, *p* < .001, CFI = .82, RMSEA = 0.06, SRMR = 0.16. These results suggest substantive differences between ethnic majority and minority groups in the variance around the intercepts and slopes. We therefore allowed the variances and covariances of the intercepts and linear slopes *between* ethnic groups to vary but constrained them to equality across the different birth cohorts within each ethnic group.

**Figure 4:**
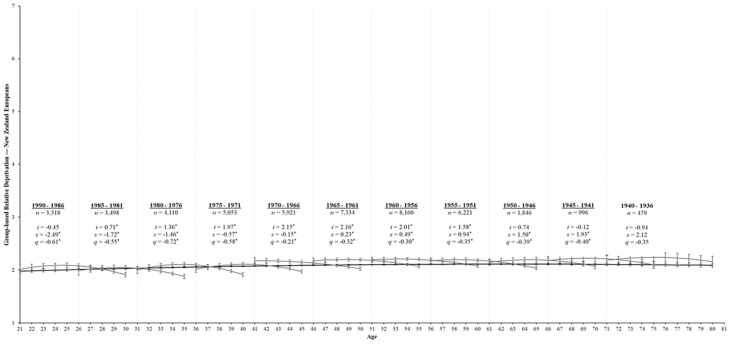
Change trajectories and comparison of aging and cohort models for ethnic majorities’ perceptions of GRD. *Note*. Change trajectories for GRD are shown by the black line from ages 21 to 80. The gray lines within each 5-year birth cohort panel demonstrate longitudinal change in GRD over 10 assessments by estimating the latent intercept (*i*), linear slopes (*s*), and quadratic slopes (*q*) and overlap with subsequent birth cohorts. Mean levels of GRD are shown on the *y*-axis across age (in years) and annual assessments on the *x*-axis with 95% confidence intervals as error bars around each point estimate. Birth cohorts with significant rates of change over time are underlined for clarity. *p < .05.

**Figure 5.**
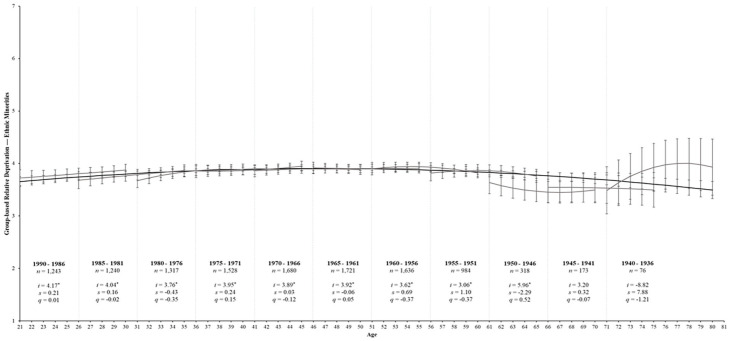
Change trajectories and comparison of aging and cohort models for ethnic minorities’ perceptions of GRD. *Note*. Change trajectories for GRD are shown by the black line from ages 21 to 80. The gray lines within each 5-year birth cohort panel demonstrate longitudinal change in GRD over 10 assessments by estimating the latent intercept (*i*), linear slopes (*s*), and quadratic slopes (*q*) and overlap with subsequent birth cohorts. Mean levels of GRD are shown on the *y*-axis across age (in years) and annual assessments on the *x*-axis with 95% CIs as error bars around each point estimate. Birth cohorts with significant rates of change over time are underlined for clarity *p < .05.

Our final model for the aging process fits these data well, χ^2^_(1419)_ = 5,680.03, *p* < .001, CFI = .94, RMSEA = 0.03, SRMR = 0.06; see Table 2. Regarding majority group members, GRD was consistently low across the lifespan, as expected for objectively advantaged groups. That said, ethnic minorities exhibited a small linear increase in GRD over time (see Table 3; *s* = 0.03, *SE* = 0.00, *p* < .001), but with a downward curve (*q* = −0.01, *SE* = 0.00, *p* < .001). Indeed, the black line depicted in Figure 4 illustrates that GRD gradually increased until around age 55 and then stabilized before gradually decreasing from around age 65 onwards. Regarding ethnic minorities, GRD exhibited curvilinear decreases over time (*q* = −0.04, *SE* = 0.01, *p* < .001). Specifically, GRD increased from age 21 to around age 45 and then steadily declined thereafter across the rest of the lifespan (see Figure 5).

The period model also fit our data well, χ^2^_(1399)_ = 5,242.61, *p* < .001, CFI = .94, RMSEA = 0.03, SRMR = 0.05; Table 2. For majority group members, Table 6 demonstrates a pattern similar to IRD, whereby the intercepts for GRD were higher for each successive birth cohort. These results suggest that older majority group members were initially higher in GRD than their younger counterparts. The period model also indicated linear decreases in GRD among ethnic majorities over time (*s* = −0.17, *SE* = 0.01, *p* < .001), albeit without curvilinear change (*q* = 0.00, *SE* = 0.00, *p* = .412).

**Table 6:** Parameter Estimates for the Period Model for Perceptions of GRD by Ethnic Group.

Birth cohort		Ethnic majority						Ethnic minority					
		Estimate	*SE*	Est./*SE*	*p*	95% CI		Estimate	*SE*	Est./*SE*	*p*	95% CI	
						LB	UB					LB	UB
1990–1986	Intercepts	1.71	0.03	56.19	<.001	1.65	1.77	4.04	0.07	57.20	<.001	3.90	4.18
1985–1981	Freely	1.76	0.02	76.65	<.001	1.71	1.80	3.94	0.06	69.03	<.001	3.83	4.06
1980–1976	Estimated	1.89	0.02	112.09	<.001	1.86	1.93	3.92	0.05	82.75	<.001	3.83	4.02
1975–1971		2.01	0.01	156.51	<.001	1.98	2.03	3.92	0.04	97.80	<.001	3.84	4.00
1970–1966		2.13	0.01	182.26	<.001	2.11	2.15	3.88	0.04	102.92	<.001	3.81	3.97
1965–1961		2.26	0.01	178.72	<.001	2.23	2.28	3.89	0.04	96.74	<.001	3.81	3.97
1960–1956		2.34	0.02	150.05	<.001	2.31	2.37	3.89	0.05	81.91	<.001	3.79	3.98
1955–1951		2.39	0.02	114.64	<.001	2.35	2.43	3.84	0.07	57.95	<.001	3.71	3.97
1950–1946		2.48	0.03	83.10	<.001	2.42	2.54	3.57	0.11	32.91	<.001	3.36	3.78
1945–1941		2.58	0.04	65.14	<.001	2.51	2.66	3.62	0.15	23.65	<.001	3.32	3.92
1940–1936		2.68	0.05	50.36	<.001	2.58	2.79	3.82	0.24	16.17	<.001	3.36	4.28
All cohorts	Linear slope constrained	–0.17	0.01	–15.30	<.001	–0.19	–0.14	0.05	0.02	2.47	.013	0.01	0.10
All cohorts	Quadratic slope constrained	0.00	0.00	0.82	.412	–0.00	0.01	–0.04	0.01	–4.34	<.001	–0.05	–0.02

*Note.* Ethnic majority = Europeans/Pākehā; Ethnic minority = Māori, Pasifika, and Asian. *SE* = standard error; CI = confidence interval; LB = lower bound; UB = upper bound. Variances were constrained to equality across birth cohorts and within ethnic groups, variance of quadratic slopes was constrained to 0. Variances for ethnic majority (*i* = 0.51*, *SE* = 0.01; *s* = 0.00, *SE* = 0.00; *q* = 0.00, *SE* = 0.00). Variances for ethnic minority (*i* = 2.04*, *SE* = 0.04; *s* = 0.16*, *SE* = 0.02; *q* = 0.00, *SE* = 0.00).

* *p* < .05.

In contrast to ethnic majorities, the intercept values for each birth cohort were generally very similar among ethnic minorities (see Table 6). Indeed, although the intercepts were consistently higher than their ethnic majority counterparts, the period model revealed no differences in initial levels of GRD between birth cohorts (i.e., the confidence intervals for the estimates largely overlapped). The one exception is the 1950 to 1946 cohort, whose intercept was lower than the other birth cohorts. The period model also indicated linear increases in GRD among ethnic minorities over time (*s* = 0.05, *SE* = 0.02, *p* = .013) but with a downward curve (*q* = −0.04, *SE* = 0.01, *p* < .001).

Finally, the cohort model also fits our data well, χ^2^_(1359)_ = 5,043.51, *p* < .001, CFI = .95, RMSEA = 0.03, SRMR = 0.05; Table 2. For the majority group members, 10 of the 11 birth cohorts showed significant change in GRD over time (*p*s < .05; see Table 7). Specifically, the youngest five cohorts showed curvilinear decreases in GRD over time, whereas older cohorts demonstrated linear increases in GRD over time with a downward curve (see Table 7). Only the oldest birth cohort (1940–1936) for ethnic majority group members demonstrated stability in GRD over time (*p*s > .05).

**Table 7.** Parameter Estimates for the Cohort Model for Perceptions of GRD by Ethnic Group.

Birth cohort		Ethnic majority					Ethnic minority				
		Est.	*SE*	*p*	95% CI		Est.	*SE*	*p*	95% CI	
					LB	UB				LB	UB
1990–1986	*i*	–0.45	0.52	.394	–1.48	0.58	4.17	0.93	<.001	2.35	5.98
	*s*	–2.49	0.56	<.001	–3.59	–1.39	0.21	0.98	.828	–1.71	2.13
	*q*	–0.61	0.15	<.001	–0.90	–0.32	0.01	0.26	.962	–0.49	0.51
1985–1981	*i*	0.71	0.28	.011	0.17	1.26	4.04	0.51	<.001	3.03	5.04
	*s*	–1.72	0.41	<.001	–2.53	–0.91	0.16	0.75	.834	–1.31	1.62
	*q*	–0.55	0.15	<.001	–0.84	–0.26	–0.02	0.27	.949	–0.54	0.50
1980–1976	*i*	1.36	0.10	<.001	1.17	1.56	3.76	0.19	<.001	3.39	4.13
	*s*	–1.46	0.24	<.001	–1.92	–1.00	–0.43	0.43	.318	–1.27	0.41
	*q*	–0.72	0.13	<.001	–0.97	–0.46	–0.35	0.23	.134	–0.81	0.11
1975–1971	*i*	1.97	0.02	<.001	1.94	2.01	3.95	0.05	<.001	3.85	4.04
	*s*	–0.57	0.09	<.001	–0.75	–0.38	0.24	0.19	.201	–0.13	0.60
	*q*	–0.58	0.11	<.001	–0.80	–0.36	0.15	0.21	.484	–0.27	0.57
1970–1966	*i*	2.15	0.02	<.001	2.12	2.18	3.89	0.04	<.001	3.81	3.97
	*s*	–0.15	0.04	<.001	–0.22	–0.08	0.03	0.07	.622	–0.10	0.16
	*q*	–0.21	0.10	.039	–0.42	–0.01	–0.12	0.20	.534	–0.50	0.26
1965–1961	*i*	2.16	0.04	<.001	2.09	2.23	3.92	0.07	<.001	3.78	4.06
	*s*	0.23	0.12	.044	0.01	0.46	–0.06	0.28	.793	–0.51	0.39
	*q*	–0.32	0.09	.001	–0.50	–0.14	0.05	0.19	.806	–0.32	0.41
1960–1956	*i*	2.01	0.11	<.001	1.80	2.22	3.62	0.23	<.001	3.16	4.08
	*s*	0.49	0.20	.013	0.10	0.87	0.69	0.44	.113	–0.16	1.55
	*q*	–0.30	0.09	.001	–0.47	–0.13	–0.37	0.20	.058	–0.76	0.01
1955–1951	*i*	1.58	0.24	<.001	1.10	2.05	3.06	0.59	<.001	1.90	4.22
	*s*	0.94	0.30	.002	0.34	1.53	1.10	0.76	.147	–0.39	2.58
	*q*	–0.35	0.09	<.001	–0.54	–0.17	–0.37	0.24	.117	–0.84	0.09
1950–1946	*i*	0.74	0.55	.178	–0.34	1.82	5.96	1.38	<.001	3.26	8.67
	*s*	1.50	0.54	.006	0.44	2.56	–2.29	1.36	.094	–4.96	0.39
	*q*	–0.39	0.13	.003	–0.64	–0.13	0.52	0.33	.117	–0.13	1.17
1945–1941	*i*	–0.12	1.09	.913	–2.25	2.01	3.20	2.77	.248	–2.23	8.63
	*s*	1.93	0.86	.025	0.24	3.61	0.32	2.20	.886	–4.00	4.63
	*q*	–0.40	0.17	.019	–0.73	–0.07	–0.07	0.43	.867	–0.92	0.78
1940–1936	*i*	–0.94	2.12	.656	–5.09	3.21	–8.82	5.68	.121	–19.95	2.32
	*s*	2.12	1.40	.131	–0.63	4.87	7.88	3.78	.037	–0.48	15.29
	*q*	–0.35	0.23	.126	–0.81	0.10	–1.21	0.62	.052	–2.43	0.01

*Note.* Ethnic majority = Europeans/Pākehā; Ethnic minority = Māori, Pasifika, and Asian. *I =* intercept; *s* = linear slope; *q* = quadratic slope. *SE* = standard error; CI = confidence interval; LB = lower bound; UB = upper bound. Variances were constrained to equality across birth cohorts and within ethnic groups, variance of quadratic slopes was constrained to 0. Variances for ethnic majority (*i* = 0.51*, *SE* = 0.01; *s* = 0.00, *SE* = 0.00; *q* = 0.00, *SE* = 0.00). Variances for ethnic minority (*i* = 2.04*, *SE* = 0.04; *s* = 0.16*, *SE* = 0.02; *q* = 0.00, *SE* = 0.00).

* *p* ≤ .05.

Conversely, all birth cohorts showed nonsignificant rates of change in GRD over time among ethnic minorities (*p*s > .05). Inspection of these nonsignificant trajectories (see Figure 5) revealed slight increases in GRD for the 1990 to 1976 birth cohorts, followed by relatively stable estimates across the 10 assessments for the 1975 to 1961 birth cohorts. The 1960 to 1951 display small increases in GRD with a downward curve, while the 1950 to 1946 and 1945 to 1941 birth cohorts display small curvilinear increases and decreases, respectively. Finally, the oldest 1940 to 1936 birth cohort displayed considerable increases over time with a downward curve. While none of these findings were statistically significant, they demonstrate some differences between minority and majority group birth cohorts in their development of GRD over time.

The aging, period, and cohort models are again similar in their fit to our data and suggest a combination of these processes contributes to changes in GRD over time. Regarding majority group members, Figure 4 suggests some subtle differences between the younger and older cohorts; younger cohorts appear to be decreasing at a greater rate over time than older cohorts. However, there is considerable overlap between estimates for both majority and minority group members (i.e., the 95% error bars overlapped; see Figures 4 and 5), and the trends for each cohort model followed the aging trend line (the black line), particularly among minority group members. Thus, while our results demonstrate status-based differences in the trajectory of GRD over time (i.e., Hypothesis 2b), the development of GRD among both majority and minority group members appears to reflect a normative aging process across adulthood rather than cohort-based effects.

## Discussion

The current research utilized cohort-sequential latent growth modeling to examine the longitudinal trajectories of IRD and GRD across the adult lifespan. In doing so, we are the first to investigate whether changes in relative deprivation over time reflect a normative aging process or generational differences. We also inspected whether the trends for IRD and GRD differed by group status (i.e., majority or minority group membership). Given that IRD and GRD reflect interpersonal and intergroup comparisons, respectively (see Smith et al., 2012), we expected distinct trajectories for IRD and GRD across the lifespan. We describe our hypotheses and results below.

### Individual-Based Relative Deprivation

We expected IRD to exhibit a curvilinear U-shaped trajectory whereby IRD decreased from young to middle adulthood and then increased thereafter (Hypothesis 1a). Our results partially support this hypothesis; mean levels of IRD declined from ages 21 to 80, although this rate of decline only decelerated (instead of reversing direction) in late adulthood for majority group members. Conversely, minority group members only showed a *linear* decrease from ages 21 to 80. That mean levels of IRD declined across the adult lifespan corroborates past work showing that *objective* income and wealth accumulation occurs as people enter and gain experience in the workforce (e.g., Jappelli, 1999; Lim & Zeng, 2016). Our results also mirror research suggesting that social comparisons are highest in young adulthood and decline thereafter (Buunk et al., 2020; Callan et al., 2015).

Indeed, the decline in feelings of IRD across the lifespan may reflect a general reduction in social comparison tendencies as people age and find social comparisons less relevant for determining their *individual* societal position (Buunk et al., 2020). In fact, as people age, *temporal* comparisons to oneself become more prevalent (Suls, 1982, 2014). While we expected an increase in mean levels of IRD at older ages (as people leave the workforce; see Hypothesis 1a), the decelerating rate of decline in IRD in older adulthood for majority group members may reflect this shift from social to temporal comparisons across the lifespan. Moreover, this trajectory may reflect a standstill in wealth accumulation as people enter retirement. While examining the extent to which the developmental trajectory of IRD reflects age differences in comparison processes—and one’s objective material conditions—is beyond the scope of the present study, future research should consider these factors when examining how IRD changes across time.

With respect to cohort effects, our results show a change in IRD over time across nine (out of 11) birth cohorts for the majority group and six birth cohorts for minority groups. For both majority and minority group members, younger cohorts started at higher levels of IRD and demonstrated steeper declines in IRD than their older counterparts. These results corroborate research showing that younger generations experience a unique socioeconomic context relative to their older counterparts (e.g., Kontopantelis et al., 2018). Nevertheless, there was considerable overlap in mean levels of IRD between birth cohorts, suggesting that changes in IRD over time are explained mainly by a normative aging process rather than cohort-based effects (i.e., Hypothesis 1a). That the trajectory for IRD was similar for ethnic majority and minority groups (albeit with higher mean levels of IRD among ethnic minorities) further supports Hypothesis 1b and indicates that mean levels of IRD change over time *regardless* of ethnic group membership. In other words, IRD stems from inter*personal* rather than inter*group* comparisons over time (Smith et al., 2012).

### Group-Based Relative Deprivation

Contrary to IRD, we expected trajectories of GRD to differ across cohorts based on the sociopolitical context in which one was born and grew up (Hypothesis 2a). Likewise, we expected this trajectory to differ by ethnic status (Hypothesis 2b). But contrary to Hypothesis 2a, GRD followed a normative aging process over time rather than cohort-based effects. Indeed, while 10 out of the 11 birth cohorts among ethnic majorities demonstrated a change in GRD over time, there were only subtle differences, and estimates overlapped considerably between birth cohorts. Instead, our aging model of majority group members revealed small increases in GRD from age 21 until age 55 before stabilizing, with slight decreases in GRD in late adulthood. Likewise, none of the ethnic minority birth cohorts displayed a significant change in GRD over time, although our aging model demonstrated higher mean levels of GRD and more pronounced curvilinear change over time (relative to ethnic majorities), with increases in GRD from age 21 to around age 45 before declining across the remaining lifespan. These results conflict with our birth cohort hypotheses (Hypothesis 2a) and suggest different GRD trajectories over time between ethnic groups (Hypothesis 2b).

While unexpected, there are some potential explanations for this developmental trajectory. For example, the increase in GRD from young-to-middle adulthood suggests that intergroup competition and comparisons may increase as people experience normative changes in their economic conditions (i.e., changes in career momentum or aspirations; see Peterson et al., 2020; Zubielevitch et al., 2023). Perhaps more importantly, these results may reflect the increased importance and salience attached to one’s ethnic identity as people age (Fadjukoff & Kroger, 2016; Maehler, 2022). The subsequent “standstill” in GRD at older ages of the lifespan among majority group members may, in turn, reflect an achieved, stable ethnic identity (and, thus, GRD). Likewise, the *decline* in GRD among older minority group members suggests that the fiscal position of one’s ethnic group in society is more relevant to younger ethnic minorities whose economic disadvantages (e.g., Marriott & Sim, 2015)—and, thus, ethnic identity—may be most salient. Given the longitudinal associations between group identification and GRD (see Zubielevitch et al., 2020), future research should consider how these two constructs may develop in tandem across the lifespan.

That our results for ethnic minorities revealed no significant cohort-based differences is somewhat surprising, given that GRD stems from intergroup comparisons that are often intrinsically linked with specific intergroup contexts (Duckitt & Mphuthing, 2002; Grant & Brown, 1995; Smith et al., 2012). These findings may help explain why collective responses to inequality are exceedingly rare (Jost et al., 2017). Perceptions of—and anger toward—injustice are integral to collective action and protest support (Jost et al., 2017; Osborne et al., 2019; Smith et al., 2012; Thomas et al., 2020). If ethnic minorities’ perceptions of fiscal relative deprivation do not differ by birth cohort, the stability in perceptions of inequality across generations may explain why we fail to see sustained efforts to redress economic inequalities (e.g., Laurin et al., 2013). That said, sample size requirements for our analyses led us to combine Māori, Pasifika, and Asian participants into a single pan-ethnic minority group, which may overlook significant sociopolitical differences among different ethnic groups. We therefore caution against assuming that one’s generational sociopolitical context does not affect how one experiences and perceives inequality and encourage future research to explore generational differences in perceived inequalities among different ethnic minority groups.

### Implications of Findings for Understanding Perceptions of Inequality

While our results illustrate the development of IRD and GRD across the lifespan, they also offer insights into how individual- and group-based perceptions of inequality may develop more broadly. Namely, IRD decreased across the lifespan while GRD increased, suggesting a “personal/group” discrepancy across the lifespan whereby people do not perceive their group and themselves as deprived simultaneously (Crosby, 1984; D. M. Taylor et al., 1990). While such discrepancies are well-established in research assessing perceived *discrimination* (Crosby, 1984; Operario & Fiske, 2001), the extent to which people can feel both personally and collectively deprived remains relatively underexplored (for exceptions, see Foster & Matheson, 1995; Osborne, Sibley, Smith, & Huo, 2015), and research has yet to document this phenomenon across the lifespan. Our results contribute to this research by demonstrating how IRD and GRD trajectories differ across adulthood.

Our results also provide essential insights into the life stages where feelings of relative deprivation are most prevalent and, thus, reveal the age groups whereby adverse health, well-being, and intergroup biases may be most salient. Indeed, younger individuals and birth cohorts generally report higher feelings of IRD, which helps explain *why* these age groups report poorer mental health outcomes (e.g., Twenge et al., 2019). Likewise, that GRD increases until middle adulthood may explain the “crystallization” of racial prejudices that ends in this life stage (Henry & Sears, 2009). That is, feelings of GRD and intergroup biases may be most malleable in younger years and relatively stable thereafter (see Alwin & Krosnick, 1991; Krosnick & Alwin, 1989; Osborne et al., 2011). If feelings of GRD increase from young to middle adulthood, targeting these life stages may be most fruitful for reducing racial prejudices, particularly among ethnic majorities. Future research should consider these possibilities.

### Strengths, Caveats, and Future Directions

The present study is the first to examine the trajectory of IRD and GRD across the adult lifespan. Indeed, our use of longitudinal panel data from a large, nationwide random sample—coupled with theoretically relevant measures of relative deprivation (see Smith et al., 2012)—allows us to chart how feelings of IRD and GRD change over time. Specifically, using a cohort-sequential design allows us to directly compare age, period, and cohort effects over time (Prinzie & Onghena, 2005). Our unprecedented sample size also allows us to compare ethnic minority and majority groups and identify how the development of relative deprivation differs by *objective* group status. Our study therefore uniquely disentangles these processes and offers novel insights into how relative deprivation changes as we age, as well as the potential generational and status-based differences over time.

Nevertheless, future research is needed to identify the mechanisms and moderators of our results. Indeed, understanding *why* IRD decreases as people age—or why GRD increases from young to middle adulthood—is integral to understanding how and when people will respond to inequality. While we argue that changes in one’s comparison processes or material conditions affect IRD’s development over time (and the relationship between identity development and GRD), future research is needed to see if these factors shape the development of IRD and GRD across the lifespan. Likewise, future research should investigate whether similar trajectories emerge in adults younger (and older) than our sample. Indeed, feelings of IRD may differ for those just entering adulthood (i.e., age 18) or those in older adulthood (i.e., those older than 80). Finally, feelings of relative deprivation, particularly IRD, may differ by gender or socioeconomic status (SES). Exploring these factors was outside the scope of this study, as the proportion of women in each birth cohort varied considerably (see Table 1), and changes in SES are unlikely to be captured using 1-year intervals. Further investigations incorporating these variables are needed to paint a more detailed picture of relative deprivation across the adult lifespan.

In addition, while our measures of IRD and GRD were theoretically relevant and encompassed both the affective and cognitive components of relative deprivation (see Smith et al., 2012), both measures were mean scores of two items. Accordingly, we could not examine measurement invariance among our constructs longitudinally and between ethnic groups. Likewise, the associations documented here are likely attenuated due to the measurement error inherent in observed (vs latent) variables. Future research should utilize measures with more than two items to account for measurement error among the constructs (see Griliches & Hausman, 1986). More generally, computational restrictions required us to estimate our models for IRD and GRD separately. These constructs, however, are correlated, with some research suggesting that IRD *precedes* GRD (Lilly et al., 2023; Pettigrew, 2002). Future research should therefore examine whether IRD influences the development of GRD over time.

Finally, our measures of IRD and GRD encompassed perceptions of *fiscal* relative deprivation. Future research should examine whether similar trajectories emerge for non-economic forms of relative deprivation (e.g., discrimination or other resource-based relative deprivation). For example, forms of relative deprivation that focus on intergroup *treatment* may demonstrate more generational differences due to societal shifts toward “modern” and “subtle” forms of racism (e.g., Carson Byrd, 2011; Pettigrew & Meertens, 1995). While fiscal relative deprivation is an important psychological construct, examinations of other subjective assessments of status, power, and privilege (or lack thereof) in society are necessary to fully elucidate how relative deprivation develops and changes over time (and, if so, in which domains). That said, relative deprivation and social evaluations across all domains require similar cognitive and affective processes (Crosby, 1976; Runciman, 1966; Suls, 2014). Accordingly, the average effect sizes for different forms of relative deprivation should be similar (Smith et al., 2012), suggesting that the relationships between relative deprivation and outcomes are a product of the comparison process rather than a particular *domain* of social evaluation. Nonetheless, the trajectories of other forms of relative deprivation are worthy of exploration, and our results provide crucial foundations for future investigations into the development of relative deprivation across the lifespan.

## Conclusion

Despite the known effects of relative deprivation across myriad domains, research has yet to examine how feelings of IRD and GRD develop and change over time. The current study addressed this oversight by comparing aging, period, and cohort effects in IRD and GRD across the adult lifespan (ages 21–80) using a cohort-sequential latent growth design. Although IRD and GRD displayed distinct trajectories, both generally followed a normative aging process. Specifically, feelings of IRD decreased over the adult lifespan with few differences between ethnic minority and majority groups. However, the development of GRD differed between ethnic groups, with ethnic minorities experiencing greater curvilinear changes in GRD across the adult lifespan. Together, our results provide nuanced insights into how feelings of relative deprivation change across the lifespan and offer a springboard for future longitudinal research investigating how people perceive and respond to inequality.

## Notes

1. We focus on fiscal measures of relative deprivation due to their relevance to objective economic conditions.
